# Multidisciplinary approach in diagnosis and treatment of Fong disease

**DOI:** 10.1093/jscr/rjae207

**Published:** 2024-04-08

**Authors:** Nia A Gecheva, Valentin Angelov, Radka Kaneva, Milka Dikova

**Affiliations:** Department of Orthopedics and Traumatology, Medical University Sofia, Sofia 1431, Bulgaria; Department of Pediatric Orthopaedics and Hand Surgery, University Hospital of Orthopedics “Prof.B. Boichev”, Sofia, Bulgaria; Department of Orthopedics and Traumatology, Medical University Sofia, Sofia 1431, Bulgaria; Department of Pediatric Orthopaedics and Hand Surgery, University Hospital of Orthopedics “Prof.B. Boichev”, Sofia, Bulgaria; Department of Medical Chemistry and Biochemistry, Medical Faculty, Molecular Medicine Center, Medical University Sofia, Sofia 1431, Bulgaria; Laboratory of Genomic Diagnostics, Department of Medical Chemistry and Biochemistry, Medical Faculty, Medical University Sofia, Sofia 1431, Bulgaria; Department of Orthopedics and Traumatology, Medical University Sofia, Sofia 1431, Bulgaria; Department of Pediatric Orthopaedics and Hand Surgery, University Hospital of Orthopedics “Prof.B. Boichev”, Sofia, Bulgaria

**Keywords:** Fong disease, patella, genetic, diagnostics

## Abstract

Nail-patella syndrome, also known as Fong disease, is an uncommon autosomal dominant disorder characterized by a distinctive set of features, including fingernail abnormalities, hypoplastic patellae, radial head dislocation, and iliac horns. This condition often leads to patellar subluxation or dislocation, resulting in knee instability and pain. While existing literature predominantly focuses on the clinical and radiological aspects of nail-patella syndrome-related knee manifestations, only a limited number of articles delve into a meticulous approach to the condition with a comprehensive strategy for diagnosis. We present an atypical case of Fong disease distinguished by unique genetic characteristics and subsequently subjected to a thorough clinical assessment and a meticulously planned operative treatment regimen.

## Introduction

Hereditary onycho-osteodysplasia (HOOD) or nail-patella syndrome (NPS) is a hereditary condition that affects both ectodermal and mesodermal structures. The condition is autosomal dominant and is caused by heterozygous loss-of-function mutations in the LMX1B gene [1, 2]. LMX1B belongs to the LIM-homeobox transcription factor family, and its crucial function lies in orchestrating the normal patterning of the dorsoventral axis in limb and kidney development. The clinical findings include hypoplastic or absent patella, dystrophic nails, dysplasia of the elbows, usually involving radial head subluxation, and iliac horns. Complaints of the knee are reported in up to 74% of patients with NPS. In 40% of cases—nephropathy and glaucoma are the most severe complications of this syndrome. The study endeavors to unravel the intricate manifestation of the disease, delving into its clinical, radiological, and genetic nuances. Additionally, it crafts a treatment protocol, weaving together the threads of orthopedic and pediatric care.

## Case presentation

The presented patient is a 4-year-old female, born from a second normal pregnancy. She showed adequate psychomotor and neuropsychological development. She had no complaints of pain in her knees or hands. Her birth history was not significant, and she had no other medical or surgical history. At 11 months of age, the child is reported to have started walking on toes. This distinctive gait is prompt in the present day.

The family anamnesis includes a wide range of connective tissue disorders closely linked to HOOD (Fong disease). The father is reported to have varicose veins and hypermetropia; grandmother—operated from inguinal hernia and grandfather—psoriatic arthritis.

**Figure 1:**
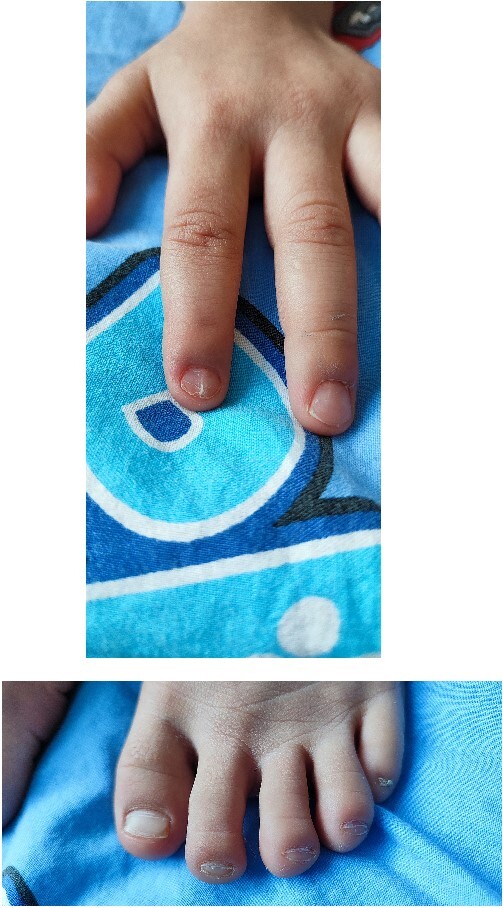
Paper-thin nails—both on hand and foot.

In 2022, the child was hospitalized in an orthopedic department as surgical treatment was indicated due to a patellar dislocation. The clinical findings included a height of 99 cm, a weight of 12 kg, paper-thin nails with longitudinal striations on the second finger on both hands and grooved, with abnormal lunules, thin nails on all toes on both feet (Fig. 1).

The respiratory system showed no significant divergence. The cardiovascular system presented with a systolic murmur, without propagation, with a minute frequency of 100 beats.

**Figure 2:**
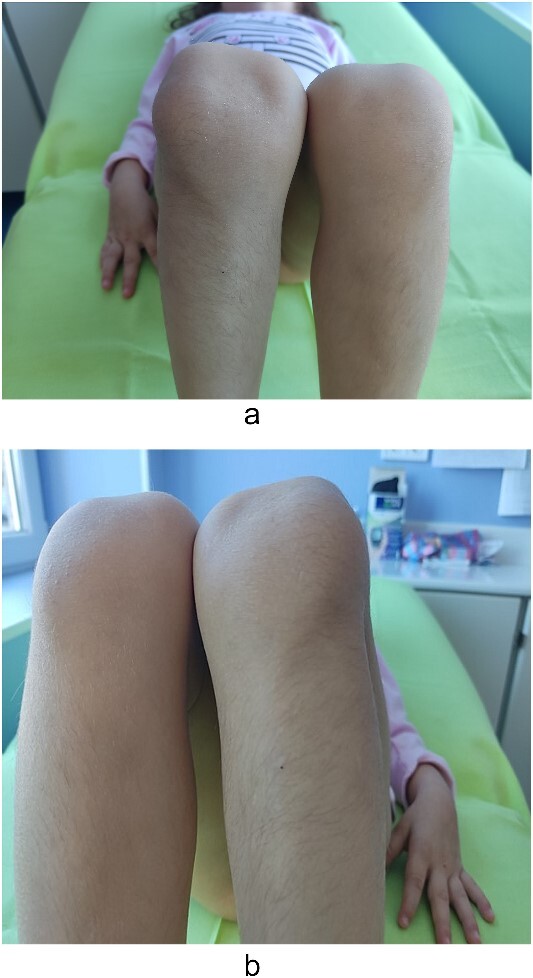
Lateralization of patella—clinical findings.

Examination of the right and left upper limbs revealed slightly underdeveloped arm muscles, but no deficiency in motor function was noticed, neither radial head dislocation. Lateral dislocation of the patella with every flexion and relocation, with extension, was observed. Normal range of motion was discovered for the left knee; the patella was hypoplastic and had unstable kinematics (Fig. 2). No pain was elicited with patellar movements, and the knees were otherwise stable. Severe spine deformity was not present except for slightly distinguishable lordosis. Examination of the abdomen, central nervous system (CNS), and fundus was normal.

### Genetic analysis

**Figure 3:**
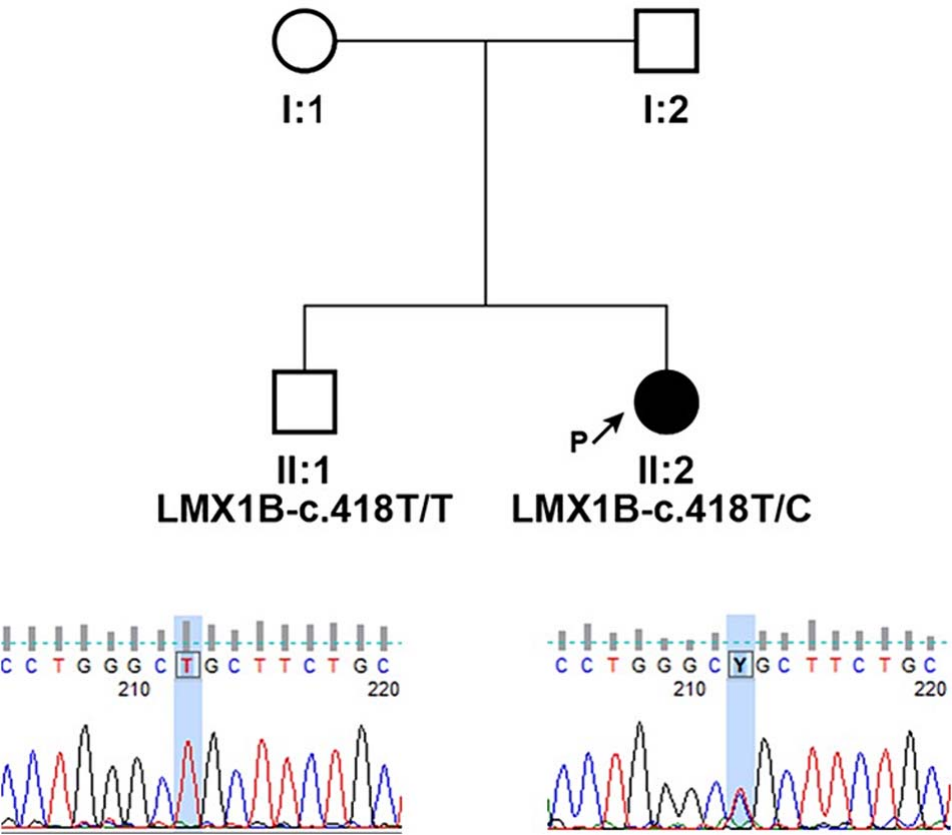
Pedigrees of presented family and segregation analysis of identified c.418 T > C (p.Cys140Arg) in LMX1B; individuals are identified by pedigree number; squares indicate males, circles indicate females, solid symbols indicate affected individuals, open symbols indicate unaffected individuals, and black arrow indicates the proband; sequencing chromatograms showing mutation segregation in the healthy brother is presented.

Peripheral blood samples of the proband and her brother were collected for a molecular genetic analysis, and DNA was extracted from leukocytes using the Chemagic DNA blood 10 k kit H1 and the Chemagen Magnetic Separation Module. Targeted resequencing was performed using the CCD17 sequencing panel. The pathogenicity of novel single nucleotide variants (SNV) was predicted by PolyPhen-2, SIFT (http://sift.bii.a-star.edu.sg/), MutationAssessor, and FATHMM software. The pathogenicity of variants was ascertained according to the criteria of the American College of Medical Genetics (ACMG), which classify variants according to five categories (benign, likely benign, uncertain significance, likely pathogenic, and pathogenic). The pedigree of the family is shown in Fig. 3. Genetic testing of the proband’s DNA identified a novel variant c.418 T > C (p.Cys140Arg) in the gene LMX1B. This variant has not been reported in the clinic literature (ClinVar, HGMD) and is not present in the referent population (gnomAD and dbSNP). The cysteine residue at this position is highly conserved between species, and all bioinformatics programs used to assess pathogenicity predict the c.418 T > C (p.Cys140Arg) change to be pathogenic.

**Figure 4:**
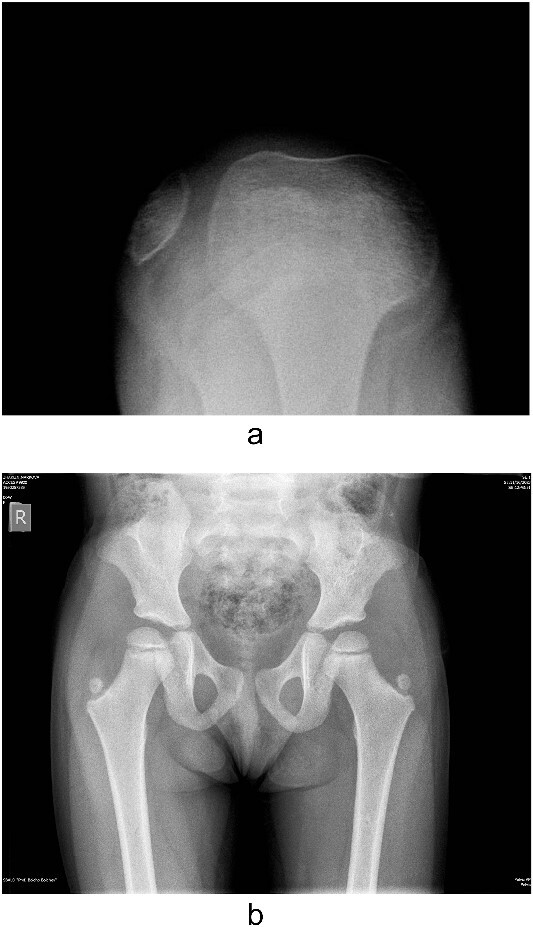
Lateralization of the patella in axial view and iliac horns.

Several radiographs were taken, showing the pathognomonic bilateral iliac horns [3], normal-shaped, without signs of hypoplasia, radio-humeral joint. Both patellae were hypoplastic, with complete lateral dislocation of the right patella, as distinguished in the skyline Merchant view (Fig. 4).

Echocardiography shows changes in the Eustachian valve and an additional chord in the left ventricle.

A renal ultrasound scan revealed normal kidneys bilaterally for site, size, and shape, and other biochemical tests yielded results within the normal range. Due to the possible association of NPS with glaucoma, the patient was referred for examination of the vision, with an unremarkable outcome.

Operative treatment: a midline parapatellar skin incision is made to expose the quadriceps mechanism and the patellar tendon and to release the lateral retinaculum and lateral femuropatellar ligament. Medially, the proximal vertical muscle fibers of vastus medialis are dressed distally, and from vertical, they become horizontal. The lateral part of the patellar tendon is detached from its tibial insertion, split from the remaining tendon, and transferred medially under the remaining half, and reattached to the periosteum, providing distal and medial advancement. The uniqueness and prerogative of the surgical technique is the establishment of both stability and no growth-blockage, as the physis remains uninfluenced.

## Discussion

NPS was first recognized as an inherited disorder by Little [4], who reported a four-generation pedigree with 18 affected members who had absent patellae and thumb nails. In 1934, Aschner [5] considered that probably four genes were involved, one for each facet of the tetrad, and that these genes were very closely linked. Renwick (1956), in a further survey of seven families, produced evidence to show that variations in the severity of the syndrome are due to modifying genes at the same locus as the main gene. The simple, dominant transmission of the condition is shown by the fact that generally affected persons have an affected parent [6] with the same blood type, but no specific blood type has a higher rate of carrying the syndrome. The underlying genetic defect has been recently localized on the distal end of the long arm of chromosome 9 in the region 9q34.1 as a mutation in the LMX1B gene [7]. The gene plays an important role not only in determining dorso-ventral patterning of the soft tissue but also in patterning of the nails, patella, and long bones during growth development [8]. Тhe patient presented in the current study is found to have the genetic version LMX1B—c.418TC(p.Cys140Arg) [9]. The degree of nail disorder varies from dysplasia and hypoplasia to almost complete absence of the nails. Turner [7] and Louboutin [10] have both discovered that thumbs are usually most affected, and the condition is becoming gradually less distinct across the hand toward the little finger. The patient reported in the case presents with nail dystrophia, predominantly affecting the thumb and index fingers.

In 90% of the cases with NPS, there is patellar instability due to a small patella, hypoplasia of the femoral condyles, or subluxation. A British study of 123 patients revealed no symptoms and normal patellar size in 16% of the patients, 75% were hypoplastic, and in 9%, no patella was found to be palpable. The unfolding of the clinical features in our observing patient included: lateralization of the patella when flexion up to 90° is applied; a deficit of the vastus medialis; and femoral condyle dysplasia. The most commonly used procedures involved patellar realignment surgery [11, 12]. The number of procedures per study ranged from 1 to 20, and they involved both proximal and distal realignment surgery. With follow-ups of 4–24 years, results were generally good. The operative technique incorporated advancement of the vastus medialis, lateral release of the retinaculum, and splitting and transfer of the ligamentum patellae proprium. No subsequent complications in the 2-years follow-up were registered.

Eye involvement is documented in ~50% of the affected individuals, and in 60%, the most considerable component of NPS is nephropathy. It usually develops in early childhood or adolescence, with accentuation of the importance of the LMX1B gene and its responsibility for the maintenance of a properly structured actin cytoskeleton and full differentiation of podocytes [13, 14]. In 15% of cases, the nephropathy manifests as chronic, benign microhematuria, and proteinuria. Approximately, 30% of cases progress to end-stage renal disease [15]. Notably, the trajectory of nephropathy follows a characteristically unforeseeable course. The patient’s documentation revealed no bothersome abnormality regarding the kidney function, and no diagnostic biopsy was recommended.

To our knowledge, the variant p.Cys140Arg, discovered in the Laboratory of Genomic Diagnostics, Department of Medical Chemistry and Biochemistry, Medical Faculty, Medical University Sofia, Bulgaria, has not been reported in patients with NPS (ClinVar, HGMD), but other variants affecting the same codon, p.Cys140Tyr and p.Cys140Phe, have already been reported as pathogenic in patients with NPS (PMID: 20531206, 11668639). According to the ACMG classification, c.418 T > C (NM_002316.4) was classified as likely pathogenic because of: (i) absence in healthy individuals (PM2), (ii) absence in the healthy sibling (PP1), (iii) occurrence in a gene that has a low rate of benign missense variation (PP2), (iv) computational tools support a deleterious effect on gene product (PP3), and (v) phenotype and family history specific for AD disease with a single gene etiology (PP4).

As the skeletal deformities inherently pose minimal hindrances to normal functioning, the significance of identifying this syndrome primarily resides in its connection to nephropathy. Consequently, early recognition of this entity becomes imperative, warranting its inclusion in the differential diagnosis for infants.

## Conclusion

HOOD—Fong disease—is regarded as an intriguing clinical phenomenon. The presented report provides a comprehensive overview of NPS, tracing its historical recognition as an inherited disorder to contemporary genetic insights. The documentation of a novel genetic variant, coupled with its classification as likely pathogenic according to ACMG guidelines, highlights the ongoing efforts in genetic diagnostics. Early recognition is vital, especially given the need for ongoing monitoring of potential nephropathy and the impact of skeletal abnormalities on the patient’s quality of life.

## References

[1] McIntosh I , Dreyer SD, Clough MV. Mutation analysis of LMX1B gene in nail-patella syndrome patients. Am J Hum Genet 1998;63:1651–8. 9837817 10.1086/302165 PMC1377636

[2] Lazzeri S , Nori G, Matocci GP. Hereditary osteo-onychodysplasia or nail-patella syndrome: description of one case and literature review. J Orthopaed Traumatol 2005;6:105–9. 10.1007/s10195-005-0092-7.

[3] Fong EE . Iliac horns (symmetrical bilateral central posterior iliac processes). Radiology 1946;47:517. 20274622 10.1148/47.5.517

[4] Little EM . Congenital absence or delayed development of the patella. Lancet 1897;II:781.

[5] Aschner B . A typical hereditary syndrome: dystrophy of the nails, congenital defect of the patella and congenital defect of the head of the radius. JAMA 1934;102:2017–20.

[6] Renwiok JH , Lawler SD. Genetical linkage between the ABO and nail-patella loci. Ann Hum Genet Lond 1966;19:312–31. 10.1111/j.1469-1809.1955.tb01356.x 14388536

[7] Turner JW . An hereditary arthrodysplasia associated with hereditary dystrophy of the nails. JAMA 1933;100:882–4. 10.1001/jama.1933.02740120020008.

[8] Brixey AM Jr , Burke RM. Arthro-onychodysplasia; hereditary syndrome involving deformity of head of radius, absence of patellas, posterior iliac spurs, dystrophy of finger nails. Am J Med 1950;8:738–44. 10.1016/0002-9343(50)90098-2. 15419189

[9] Dreyer SD , Zhou G, Baldini A, et al. Mutations in LMX1B cause abnormal skeletal patterning and renal dysplasia in nail patella syndrome. Nat Genet 1998;19:47–50. 10.1038/ng0598-47. 9590287

[10] Louboutin L , Wascher D, Neyret P. Management of patellar problems in skeletally mature patients with nail–patella syndrome. Knee Surg Sports Traumatol Arthrosc 2016;25:3012–6. 26872454 10.1007/s00167-016-4044-y

[11] Guidera KJ , Satterwhite Y, Ogden JA, et al. Nail patella syndrome: a review of 44 orthopaedic patients. J Pediatr Orthop 1991;11:737–42. 1960197

[12] Tigchelaar S , Lenting A, Bongers EMHF, et al. Nail patella syndrome: knee symptoms and surgical outcomes. A questionnaire-based survey. Orthop Traumatol Surg Res 2015. 10.1016/j.otsr.2015.09.033. 26596417

[13] Lemley KV . Kidney disease in nail-patella syndrome. Pediatr Nephrol 2009;24:2345–54. 10.1007/s00467-008-0836-8. 18535845 PMC2770138

[14] Cil1 O , Perwad F. Monogenic causes of proteinuria in children. Front Med (Lausanne) 2018;5:55. 29594119 10.3389/fmed.2018.00055 PMC5858124

[15] Bongers EM , Huysmans FT, Levtchenko E, et al. Genotype-phenotype studies in nail-patella syndrome show that LMX1B mutation location is involved in the risk of developing nephropathy. Eur J Hum Genet 2005;13:935–46. 15928687 10.1038/sj.ejhg.5201446

